# Contingency Awareness Shapes Acquisition and Extinction of Emotional Responses in a Conditioning Model of Pain-Related Fear

**DOI:** 10.3389/fnbeh.2015.00318

**Published:** 2015-11-27

**Authors:** Franziska Labrenz, Adriane Icenhour, Sven Benson, Sigrid Elsenbruch

**Affiliations:** Institute of Medical Psychology and Behavioral Immunobiology, University Hospital Essen, University of Duisburg-EssenEssen, Germany

**Keywords:** contingency awareness, pain-related fear, safety learning, fear conditioning, extinction, visceral pain, chronic pain

## Abstract

As a fundamental learning process, fear conditioning promotes the formation of associations between predictive cues and biologically significant signals. In its application to pain, conditioning may provide important insight into mechanisms underlying pain-related fear, although knowledge especially in interoceptive pain paradigms remains scarce. Furthermore, while the influence of contingency awareness on excitatory learning is subject of ongoing debate, its role in pain-related acquisition is poorly understood and essentially unknown regarding extinction as inhibitory learning. Therefore, we addressed the impact of contingency awareness on learned emotional responses to pain- and safety-predictive cues in a combined dataset of two pain-related conditioning studies. In total, 75 healthy participants underwent differential fear acquisition, during which rectal distensions as interoceptive unconditioned stimuli (US) were repeatedly paired with a predictive visual cue (conditioned stimulus; CS^+^) while another cue (CS^−^) was presented unpaired. During extinction, both CS were presented without US. CS valence, indicating learned emotional responses, and CS-US contingencies were assessed on visual analog scales (VAS). Based on an integrative measure of contingency accuracy, a median-split was performed to compare groups with low vs. high contingency accuracy regarding learned emotional responses. To investigate predictive value of contingency accuracy, regression analyses were conducted. Highly accurate individuals revealed more pronounced negative emotional responses to CS^+^ and increased positive responses to CS^−^ when compared to participants with low contingency accuracy. Following extinction, highly accurate individuals had fully extinguished pain-predictive cue properties, while exhibiting persistent positive emotional responses to safety signals. In contrast, individuals with low accuracy revealed equally positive emotional responses to both, CS^+^ and CS^−^. Contingency accuracy predicted variance in the formation of positive responses to safety cues while no predictive value was found for danger cues following acquisition and for neither cue following extinction. Our findings underscore specific roles of learned danger and safety in pain-related acquisition and extinction. Contingency accuracy appears to distinctly impact learned emotional responses to safety and danger cues, supporting aversive learning to occur independently from CS-US awareness. The interplay of cognitive and emotional factors in shaping excitatory and inhibitory pain-related learning may contribute to altered pain processing, underscoring its clinical relevance in chronic pain.

## Introduction

As a translational model in the neurosciences, fear conditioning is increasingly implemented in the field of pain research. One important argument in support of applying human fear conditioning in the context of pain is the high comorbidity of chronic pain with anxiety disorders (Breivik et al., 2006), suggesting shared mechanisms underlying both, pathological fear and pain. Indeed, altered fear learning has been demonstrated in various patient groups with chronic pain, including fibromyalgia, chronic back pain, chronic tension-type headaches and irritable bowel syndrome (IBS; Vlaeyen, 2015). These converging findings support that conditioned pain-related fear may play a role in the transition from acute to chronic pain as well as in the maintenance of chronic pain, as postulated by fear-avoidance models (Vlaeyen, 2015). Moreover, extinction-based treatment approaches have successfully been translated into the development and application of exposure therapy for chronic pain (den Hollander et al., 2010), specifically targeting pain-related fear and maladaptive avoidance behaviors (Vlaeyen, 2015). Meanwhile, the mechanisms underlying the formation and especially the extinction of pain-related fear remain incompletely understood even in healthy individuals, calling for more experimental work.

In recent years, a number of research groups has introduced innovative conditioning paradigms with clinically relevant pain stimuli as unconditioned stimulus (US) or conditioned stimulus (CS) to capture different aspects of pain-related learning and extinction processes in healthy volunteers (Meulders et al., 2011, 2013; Pappens et al., 2012, 2015; Kattoor et al., 2013; Benson et al., 2014; Gramsch et al., 2014; Icenhour et al., 2015a) and patients with chronic pain (Meulders et al., 2014, 2015; Icenhour et al., 2015b). One aspect that has not been specifically addressed in these promising experimental approaches is the role of conscious awareness of contingencies between predictive cues (i.e., CS) and pain. In general, the acquisition of emotional memories has long been assumed to rely on the awareness of relationships between cue and outcome, operationalized as the ability to verbalize this relation (Lovibond and Shanks, 2002). Contingency awareness was accordingly conceptualized as a mediator between associative learning and the display of conditioned responses (Lovibond, 2003, 2004). In support of this notion, several human studies reliably observed conditioned responses in perceived CS valence and in physiological changes of skin conductance responses in aware subjects only, suggesting that contingency awareness is necessary for successful fear conditioning (Lovibond and Shanks, 2002; Tabbert et al., 2006, 2011; Klucken et al., 2009; Mitchell et al., 2009; Lovibond et al., 2011; Weidemann and Antees, 2012; Weidemann et al., 2013). However, others have questioned this assumption given evidence that autonomic fear responses also occur without explicit knowledge regarding contingencies (Wiens and Öhman, 2002; Smith et al., 2005; Knight et al., 2006, 2009; Schultz and Helmstetter, 2010; Raio et al., 2012).

In light of this debate and first data supporting altered contingency learning and extinction in chronic pain patients (Jenewein et al., 2013; Meulders et al., 2014; Icenhour et al., 2015b), our goal was to address the putative role of contingency awareness in shaping acquisition and extinction of conditioned emotional responses to predictive cues in a visceral pain-related conditioning model. To do so, we analyzed behavioral data in a large, pooled sample of healthy volunteers with a focus on classically-conditioned changes in perceived valence of predictive cues that were either consistently paired with visceral pain as US (i.e., pain-predictive CS^+^) or cues that were never paired with pain (i.e., CS^−^). We conducted separate analyses for conditioned emotional responses to CS^+^ and CS^−^ rather than relying solely on differential measures. The rationale was that recent evidence from the broader fear conditioning field supports that safety learning processes, induced by CS^−^ as safety cues, engage distinct brain regions (Fullana et al., 2015), suggesting a separate process which may indeed play a unique role in the context of pain (Volders et al., 2012; Jenewein et al., 2013; Meulders et al., 2014; Icenhour et al., 2015a, b; Labrenz et al., 2015). To address the putative role of contingency awareness in shaping distinct negative and positive emotional learning and extinction regarding pain and safety, we implemented a new integrative measure of contingency accuracy, and tested the hypothesis that individuals with high contingency accuracy would show more pronounced negative *as well as* positive emotional responses following acquisition. In addition, we examined whether higher contingency accuracy would result in impaired extinction of emotional responses, characterized by persisting negative and positive valence of formerly pain-predictive *as well as* safety cues after extinction in individuals with high contingency accuracy. Finally, we explored associations between contingency accuracy and valence changes and tested accuracy as a predictor of variance in pain-related negative and positive emotional learning and extinction.

## Materials and Methods

### Participants

For this analysis, behavioral data from two brain imaging studies implementing identical differential conditioning paradigms using painful rectal distensions as US were pooled (Icenhour et al., 2015a; Labrenz et al., unpublished data) resulting in a sample of *N* = 75 healthy individuals (38 women, 37 men; mean age 28.87 ± 9.6 years) for the acquisition phase. Since only one of these studies (Icenhour et al., 2015a) contained an extinction phase, the sample size was *N* = 48 (24 women, 24 men; mean age 29.87 ± 10.84 years) for extinction. Exclusion criteria for both studies were age <18 or >60 years, any known medical and psychiatric conditions or chronic medication use (except hormonal contraceptives or occasional use of over-the-counter allergy or pain medications) based on self-report. All but *N* = 7 women were on oral contraceptives. A standardized in-house questionnaire was used to exclude any symptoms suggestive of functional or gastrointestinal conditions (Lacourt et al., 2014) and all participants were tested for perianal tissue damage (i.e., painful hemorrhoids) potentially interfering with balloon placement. Pregnancy was ruled out with a commercially available urinary test on the day of the study. Any previous participation in a conditioning study was also exclusionary. Screening for current anxiety or depression symptoms was accomplished with the German version of the Hospital Anxiety and Depression Inventory using published cut-off values, i.e., ≥8 (HADS; Herrmann-Lingen et al., 2005). The study protocols were approved by the local ethics committee (University of Duisburg-Essen, Germany) and followed the Declaration of Helsinki. All participants gave informed written consent and were paid for their participation.

### Experimental Protocol

The differential conditioning protocol with visceral pain as US has previously been applied in healthy volunteers (Kattoor et al., 2013; Gramsch et al., 2014) and patients with IBS (Icenhour et al., 2015b). In brief, moderately painful rectal distensions, accomplished with a pressure-controlled barostat system (modified ISOBAR 3 device, G & J Electronics, ON, Canada), served as clinically relevant and effective visceral US, representing a valid and reliable experimental model for the investigation of visceral pain processing (Mayer et al., 2008; Keszthelyi et al., 2012). The stimulus intensity (i.e., distension pressure) for conditioning was initially determined based on individual rectal pain thresholds in order to ensure comparably painful US in all individuals. To do so, individualized distension pressures corresponding to perceived pain intensities between 60 and 70 on a 0–100 mm visual analog scale (VAS) were chosen for US application during conditioning. The protocol consisted of an acquisition and an extinction phase, which were each followed by VAS ratings of CS valence and contingencies (see below). Initially, participants were instructed that during the experiment, they would see visual signals and experience repeated rectal distensions but received no information regarding experimental phases or cue-outcome contingencies. During acquisition, one geometric visual symbol (CS^+^) was consistently paired with a painful rectal distension (US) while a second visual cue (CS^−^) was never followed by the US (differential delay conditioning). Overall, 32 CS were presented (16 CS^+^ and 16 CS^−^) in pseudo-randomized order with a 75% reinforcement schedule to induce uncertainty and ensure more robust conditioned responses (Kalisch et al., 2006; Sehlmeyer et al., 2009). US onset varied randomly between 8 and 12 s after CS^+^ onset and both stimuli co-terminated. Intertrial intervals (ITI) were 20 s. During extinction, 24 CS were presented (12 CS^+^ and 12 CS^−^) in pseudo-randomized order without any US presentations. Note that extinction was conducted with a subtle context change consisting of a change in CS background color in half of the participants. However, given no context-related effects on behavioral measures or skin conductance responses (Icenhour et al., 2015a), data were pooled herein.

### Valence Ratings of CS

Conditioned changes in perceived valence of previously neutral predictive cues constitute an established behavioral marker in fear conditioning, capturing learned emotional responses which are demonstrably associated with neural correlates of pain-related fear and safety learning (Kattoor et al., 2013). To quantify emotional responses to pain-predictive cues (CS^+^) and safety cues (CS^−^), participants rated CS valence using a hand-held fiber optic response system (LUMItouch^TM^, Photon Control Inc., Burnaby, BC, Canada). Specifically, participants were prompted to indicate the unpleasantness of each cue separately on a digitized +100 to −100 VAS with end points labeled as “very unpleasant” and “very pleasant”, while “neutral” (= 0) was indicated in the middle of the scale. These ratings were accomplished prior to acquisition (baseline) and immediately following acquisition and extinction.

### Contingency Awareness and Accuracy

Awareness of CS-US contingencies was assessed with digitized VAS at the conclusion of acquisition and extinction phases. Participants were prompted to indicate how often each of the cues was followed by pain on a 0–100 mm scale with end points labeled “never” and “always”. In addition, we computed a novel and integrative measure to adequately quantify the accuracy of contingency awareness. The rationale was that in differential learning paradigms, a differentiation between CS^+^- and CS^−^-related contingencies is often reported as a marker of successful contingency learning (e.g., Tabbert et al., 2011). However, the mere differentiation does not provide sufficient information on distinct influences of pain- and safety-related behavioral responses. Moreover, contingency ratings do not resemble a direct and explicit measure of accurate contingency awareness, especially during partial reinforcement schedules where an overestimation of CS^+^-US pairings would be falsely interpreted as high awareness. Therefore, VAS ratings for CS^+^- and CS^−^-US contingencies were transformed into contingency accuracy scores in percent, assigning 100% accuracy to CS^+^-US ratings of 75 on the VAS (representing the correct contingency in this study) and 100% to CS^−^-US contingency ratings of 0 (which was also the correct contingency herein), thereby providing separate but comparable measures for CS^+^- and CS^−^-related awareness and deviations from full accuracies. Hence, for an individual CS^+^-US rating of 75 mm, contingency accuracy was considered 100%, whereas ratings of either 65 mm (i.e., underestimation of real contingency) or 85 mm (overestimation of real contingency) would result in accuracy scores of 100–13.3% = 86.7%. This measure was used for comparisons of groups with high and low contingency accuracy as well as for regression analyses (see below).

### Statistical Analyses

Statistical analyses were carried out with the Statistical Package for the Social Sciences (SPSS, IBM Corp., IBM SPSS Statistics for Windows, Version 22.0. IBM Corp., Armonk, NY, USA). To initially confirm differential changes in CS valence and CS-US contingencies, analyses of variance (ANOVA) were conducted, reporting results with Greenhouse-Geisser correction for significant interactions. *Post hoc* testing was accomplished with Bonferroni correction to control for inflation of alpha values set at *p* < 0.05 due to multiple comparisons. In order to compare participants with high vs. low contingency accuracy, a median-split was conducted based on the mean accuracy score in percent and groups were compared using ANOVA followed by *post hoc* two sample *t*-tests. Correlational analyses were accomplished using Pearson’s *r*, followed by multiple regression analyses predicting valence changes after acquisition and extinction, respectively. All data are given as mean ± standard error of the mean (SEM), unless indicated otherwise.

## Results

### Changes in CS Valence after Acquisition and Extinction

**Figure 1:**
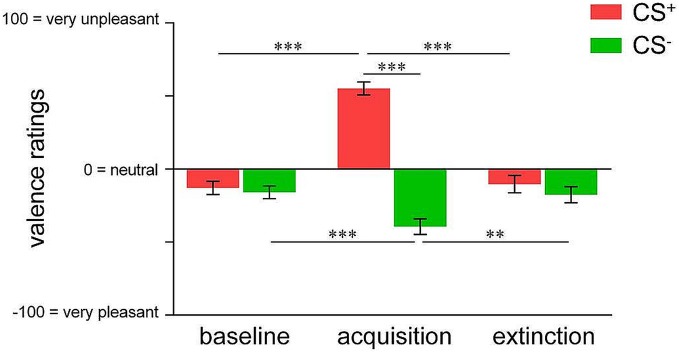
**Valence ratings of pain-predictive (CS^+^; indicated in red) and safety cues (CS^−^; indicated in green) assessed at baseline, after acquisition and extinction.** Data are shown as mean ± SEM. **p < 0.01; ***p < 0.001.

We initially confirmed differential changes in CS valence irrespective of contingency awareness for acquisition and extinction in the whole sample. For acquisition, ANOVA revealed a significant phase × CS-type interaction (*F*_(1,74)_ = 109.333, *p* < 0.001) as well as significant main effects (both *p* < 0.001). *Post hoc* *t*-tests confirmed that while CS^+^ and CS^−^ were rated as equally neutral at baseline, CS^+^ was perceived as significantly more unpleasant compared to CS^−^ following acquisition (*t*_(74)_ = 11.388, *p* < 0.001; Figure 1). Importantly, while CS^+^ unpleasantness significantly increased from baseline to after acquisition (*t*_(74)_ = 11.249, *p* < 0.001) indicating learned aversion, CS^−^ was rated as significantly more pleasant following acquisition when compared to baseline (*t*_(74)_ = 3.938, *p* < 0.001), suggestive of safety-related learning during differential conditioning (Figure 1). For extinction, a significant phase × CS-type interaction (*F*_(2,46)_ = 61.778, *p* < 0.001) as well as significant main effects (both *p* < 0.001) were found. *Post hoc* testing yielded diminished CS^+^-CS^−^ differentiation following extinction as well as significant changes of both, CS^+^ (*t*_(47)_ = 10.716, *p* < 0.001) and CS^−^ valence (*t*_(47)_ = 3.356, *p* =* 0*.002) when compared to acquisition levels, indicating successful extinction (Figure 1).

### Contingency Awareness and Accuracy

Contingency ratings regarding CS^+^-US and CS^−^-US pairings were assessed to ensure differential contingency awareness following acquisition and awareness of changed contingencies following extinction. After acquisition, perceived CS^+^-US contingency (*M* = 71.84 ± 2.53 mm) was significantly different from perceived CS^−^-US contingency (*M* = 21.11 ± 2.98 mm; *t*_(74)_ = 11.741, *p* < 0.001), confirming the formation of differential contingency awareness. After extinction, participants were aware of changed contingencies, as evidenced by comparable ratings (CS^+^-US: 11.96 ± 3.10 mm; CS^−^-US: 7.29 ± 2.10 mm).

Analysis of contingency accuracy scores revealed comparable accuracies for both the CS^+^ and the CS^−^ after acquisition (CS^+^-US: 79.89 ± 2.49%; CS^−^-US: 78.89 ± 2.98%) as well as after extinction (CS^+^-US: 88.04 ± 3.10%; CS^−^-US: 92.71 ± 2.10%). Although accuracies for both CS were comparable, they neither reached 100% after acquisition nor after extinction, indicating deviations from perfect contingency accuracies during both acquisition as excitatory and extinction as inhibitory learning. Interestingly, while acquisition CS^+^ and CS^−^ accuracy scores were not inter-correlated (*r* = 0.116; *p* = 0.321), a significant inter-correlation was found for extinction (*r* = 0.570; *p* < 0.001). This supports distinct and independent contributions of both, CS^+^ and CS^−^ processing to the formation but not the extinction of perceived CS-US contingencies.

### Analyses in Subgroups with High vs. Low Contingency Accuracy

**Figure 2:**
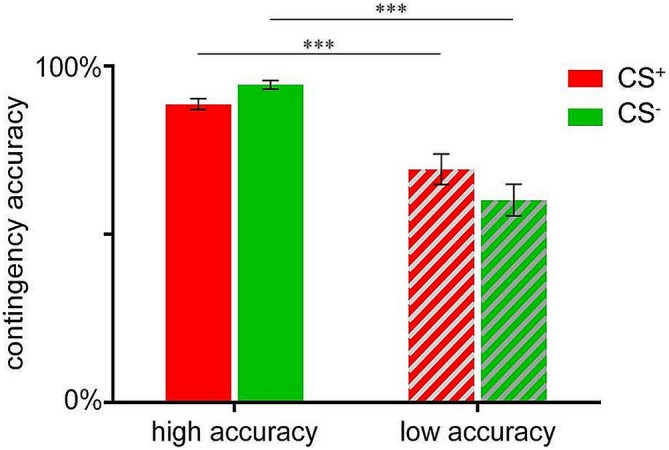
**Contingency accuracy scores for CS^+^-US (indicated in red) and CS^−^-US (indicated in green) contingencies assessed after acquisition.** Data are shown as mean ± SEM. ***p < 0.001.

In order to compare participants with high vs. low contingency accuracy with respect to valence changes (see below), a median-split was conducted based on the mean accuracy score in percent (83.33%), resulting in a group with high mean accuracy (*N* = 41; *M* = 91.54 ± 0.80%) and a low accuracy group (*N* = 34; *M* = 64.74 ± 2.81%). Note that these groups did not differ with respect to distribution of sex or age (data not shown). Independent sample *t*-tests confirmed significant group differences for both, CS^+^-US (*t*_(74)_ = 4.008, *p* < 0.001) as well as CS^−^-US contingency accuracies (*t*_(74)_ = 7.047, *p* < 0.001; Figure 2).

**Figure 3:**
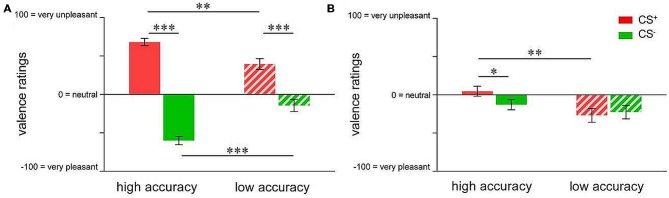
**Group differences in valence ratings of pain-predictive (CS^+^; indicated in red) and safety cues (CS^−^; indicated in green) after (A) acquisition and (B) extinction.** Data are shown as mean ± SEM. *p < 0.05; **p < 0.01; ***p < 0.001.

In order to compare the groups with respect to valence changes, repeated measures ANOVA followed by *t*-tests were conducted. For acquisition, results indicated a significant group × CS-type interaction (*F*_(1,73)_ = 26.750; *p* < 0.001). While both groups showed significant CS^+^-CS^−^ differentiation in CS valence (high accuracy: *t*_(40)_ = 14.903; *p* < 0.001; low accuracy: *t*_(33)_ = 4.537; *p* < 0.001), differential emotional responses were more pronounced in highly accurate individuals (*t*_(73)_ = 5.172; *p* < 0.001; Figure 3A). Specifically, highly accurate participants perceived the CS^+^ as more unpleasant (*t*_(73)_ = 3.348; *p* = 0.001) while the CS^−^ was rated as more pleasant (*t*_(73)_ = 4.902; *p* < 0.001), supporting enhanced emotional learning of both, danger and safety cue properties (Figure 3A). Following extinction, ANOVA of valence ratings revealed a trend towards CS-type × group interaction (*F*_(1,46)_ = 3.819; *p* = 0.057). *T*-tests yielded a significant between-group difference in CS^+^ valence (*t*_(46)_ = 2.850; *p* = 0.007), resulting from higher pleasantness in the low accuracy group (Figure 3B). Furthermore, there was a significant difference between CS^+^ and CS^−^ valence in the highly accurate group only (*t*_(24)_ = 2.124; *p* = 0.044, Figure 3B), which was attributable to persistently higher pleasantness of CS^−^ in relation to CS^+^, indicating incomplete extinction particularly of learned safety cue properties.

### Correlations and Regression Analyses

To address associations between valence ratings and contingency accuracy for the CS^+^ and CS^−^, correlation analyses were conducted (Table 1). For acquisition, analyses revealed significant correlations between CS^+^ and CS^−^ valence. Furthermore, CS^−^ contingency accuracy was significantly associated with both CS^+^ and CS^−^ valence, whereas no associations were found for CS^+^-related accuracy. For extinction, CS^+^ and CS^−^ contingency accuracy scores and valence ratings were significantly inter-correlated. Besides, significant associations between CS^+^ valence following acquisition and extinction were observed (Table 1).

**Table 1:** **Results of correlation analyses of CS valence and CS-US accuracy scores during acquisition and extinction**.

		Acquisition				Extinction			
		Accuracy		Valence		Accuracy		Valence	
		CS^+^	CS^−^	CS^+^	CS^−^	CS^+^	CS^−^	CS^+^	CS^−^
**Acquisition**
Accuracy	CS^+^	1
		75
	CS^−^	0.116	1
		0.321	75
Valence	CS^+^	0.199	0.344**	1
		0.087	**0.003**	75
	CS^−^	−0.2020	−0.559***	−0.447***	1
		0.082	**0.000**0	**0.000**	75
**Extinction**
Accuracy	CS^+^	0.156	0.2630	0.187	−0.170	1
		0.291	0.071	0.203	0.248	48
	CS^−^	−0.083	0.181	−0.019	−0.219	0.570***	1
		0.574	0.217	0.900	0.134	**0.000**	48
Valence	CS^+^	0.215	0.1000	0.482**	−0.197	0.130	0.067	1
		0.142	0.498	**0.001**	0.179	0.378	0.650	48
	CS^−^	0.112	−0.059 0	0.123	0.150	0.023	−0.124	0.479**	1
		0.450	0.692	0.405	0.310	0.879	0.399	**0.001**0	48

Significant results are indicated in bold. **p < 0.01; ***p < 0.001.

In a final step, multiple regression analyses were conducted to test if contingency accuracy constitutes a significant predictor for CS valence after acquisition or extinction. As shown in Table 2, the model for valence of CS^−^ after acquisition was predicted by CS^−^-US accuracy, along with CS^+^ valence, supporting a role of contingency accuracy in the acquisition of positive emotional responses to conditioned safety cues. The other models revealed no evidence for contingency accuracy as a significant predictor (Table 2). Together, the results suggest that explicit knowledge about CS-US relations, particularly regarding safety, is a predictor for learned positive emotional responses, while not predicting extinction of learned cue properties.

**Table 2:** **Results of multiple regression analyses with CS valence during acquisition and extinction as outcome variables and accuracy of CS-US contingencies and CS valence as predictors**.

	Outcome variable	Predictor variables	B	β	t	*p*
**Acquisition**
	**CS^+^ valence**	CS^+^-US accuracy	0.200	0.113	1.055	0.295
	*R^2^* = 0.225	CS^−^-US accuracy	0.202	0.136	1.080	0.284
	Adj. *R^2^* = 0.192***	CS^−^ valence**	−0.290	−0.348	−2.726	**0.008**
	**CS^−^ valence**	CS^+^-US accuracy	−0.204	−0.095	−1.012	0.315
	*R^2^* = 0.395	CS^−^-US accuracy***	−0.811	−0.455	−4.618	**<0.001**
	Adj. *R^2^* = 0.370***	CS^+^ valence**	−0.327	−0.272	−2.726	**0.008**
**Extinction**
		CS^+^-US accuracy	0.177	0.099	0.841	0.405
	**CS^+^ valence**	CS^−^-US accuracy	−0.117	−0.076	−0.554	0.582
	*R^2^* = 0.439	CS^+^ valence acquisition**	0.383	0.380	2.985	**0.005**
	Adj. *R^2^* = 0.372***	CS^−^ valence acquisition	−0.141	−0.154	−1.069	0.291
		CS^−^ valence extinction**	0.478	0.440	3.669	**0.001**
		CS^+^-US accuracy	0.054	0.033	0.247	0.806
	**CS^−^ valence**	CS^−^-US accuracy	0.057	0.040	0.261	0.796
	*R^2^* = 0.297	CS^+^ valence acquisition	−0.061	−0.066	−0.420	0.676
	Adj. *R^2^* = 0.213**	CS^−^ valence acquisition	0.220	0.261	1.642	0.108
		CS^+^ valence extinction**	0.508	0.551	3.669	**0.001**

Significant results are indicated in bold. **p < 0.01; ***p < 0.001.

## Discussion

The importance of pain-related fear in the pathophysiology and treatment of chronic pain is increasingly recognized, inspiring experimental work to elucidate the mechanisms underlying pain-related learning and memory processes specifically in the context of pain (Vlaeyen, 2015). Human fear conditioning studies with highly aversive or painful US support distinct emotional learning processes characterized not only by negative emotions in response to predictive danger cues (i.e., CS^+^) but also by positive emotions in response to cues signaling safety (i.e., CS^−^). At the behavioral level, these processes are reflected by changes in perceived valence of previously neutral predictive cues that turn into unpleasant or pleasant signals, respectively, depending on cue-outcome contingencies. Importantly, these learned emotional responses may be mediated by distinct neural networks (Fullana et al., 2015), and appear to be altered in patients with chronic pain (Jenewein et al., 2013; Icenhour et al., 2015b). One important, yet unsolved question concerns the putative role of conscious awareness of cue-outcome relations in shaping the acquisition and extinction of emotional responses to predictive cues. Therefore, we aimed to address the role of contingency awareness, operationalized as a novel and integrative measure of contingency accuracy, in a large, pooled sample of behavioral data from healthy volunteers undergoing visceral pain-related conditioning (Icenhour et al., 2015a; Labrenz et al., unpublished data). We hypothesized that individuals with high contingency accuracy would show more pronounced emotional responses, reflected by CS valence ratings, after conditioning. In line with this assumption, results revealed significantly more pronounced negative emotions in response to the CS^+^ as well as greater positive emotions in response to the CS^−^ in individuals with high compared to individuals with low contingency accuracy.

The findings support a role of contingency awareness in shaping distinct emotional responses to conditioned danger and safety cues. These results in healthy volunteers are in accordance with earlier evidence from our group that patients with IBS showed higher contingency awareness specifically of safety cues along with more pronounced positive emotions to the same cues (Icenhour et al., 2015b), calling for future research addressing cognitive factors in safety learning in chronic pain. To further substantiate the group differences in the present dataset, we tested if contingency accuracy was a predictor of conditioning-induced valence changes after acquisition. We found that contingency accuracy significantly predicted conditioned positive emotional responses to safety cues, suggesting a role of cognitive aspects in safety learning. On the other hand, variance in conditioned negative emotional responses to danger cues was not predicted by contingency accuracy. Hence, the acquisition of negative emotional responses to pain-related danger signals does not appear to require accurate cognitive awareness of the associative strength between cue and painful outcome. Our data therefore extend evidence from fear conditioning studies actively manipulating contingency awareness, which question the assumption of explicit knowledge about cue-outcome contingencies as a prerequisite in human aversive learning (Knight et al., 2006; Schultz and Helmstetter, 2010; Raio et al., 2012). Unlike approaches actively manipulating contingency awareness to create groups with full vs. no contingency awareness through masking (Knight et al., 2006, 2009; Weidemann et al., 2013), explicit instructions and/or distraction (Klucken et al., 2009; Schultz and Helmstetter, 2010; Tabbert et al., 2011) we herein addressed contingency accuracy developed “naturally” over the course of differential learning. While our findings may not generalize to results in individuals fully unaware of CS-US contingencies, varying manifestations of differentially acquired contingency accuracy appear closer to clinical reality in chronic pain patients. Our findings indicate that inaccurate contingencies, including over- or underestimation of associations between predictors and an expected pain-related outcome, affect learned emotional responses in a distinct manner.

Conditioning processes involving pain-related emotional learning may well play a role in the pathophysiology and/or maintenance of chronic pain (Vlaeyen, 2015). This also holds true for extinction, which although less well-studied, appears to be impaired in patients with chronic pain (Labus et al., 2013; Icenhour et al., 2015a). At the same time, extinction processes provide a framework for cognitive-behavioral treatment approaches involving exposure therapy which have been successfully tailored to the treatment of chronic pain (den Hollander et al., 2010). Therefore, understanding a putative role of cognitive factors in emotional inhibitory learning is highly relevant. We herein explored whether contingency accuracy affects the extinction of learned emotional responses, expecting persistent negative as well as positive valence of formerly predictive cues as a function of high contingency accuracy. Results of group comparisons revealed that individuals with high contingency accuracy after acquisition demonstrated persistent differential emotional responses after extinction. Interestingly, this effect was solely driven by maintenance of positive emotional responses to former safety cues, while negative emotional responses to former danger signals were fully extinguished, indicating reduced extinction particularly of positive emotional responses to cues predicting safety during acquisition. Additionally, individuals with low contingency accuracy demonstrated an unexpected reversal of previously learned emotional responses to cues formerly signaling danger, resulting in equally positive valence of both, former danger and safety signals after extinction. While these results suggest distinct processes underlying extinction of emotional responses to former danger and safety signals as well as a direct impact of contingency accuracy, regression analyses revealed no independent contribution of contingency accuracy to extinction, unlike hypothesized. Our findings rather suggest that other, possibly more complex interactions between cognitions and emotions may be involved in pain-related inhibitory learning. Nevertheless, it is conceivable that low contingency awareness and decreased cue differentiation may favor spreading of emotional attributes to the safe context of extinction, characterized by the absence of any aversive event, as a possible mechanism underlying overgeneralization with detrimental long-term effects in anxiety disorders (Lissek et al., 2010) as well as chronic pain (Jenewein et al., 2013; Meulders et al., 2014). Furthermore, persistent safety cue properties following extinction irrespective of contingency accuracy suggest a resistance especially of learned safety to inhibitory learning, thereby potentially interfering with extinction-based treatment approaches (Volders et al., 2012).

Together, our findings clearly favor a separate consideration of conditioned responses to danger and safety cues. The analysis of differential measures alone, as common practice in fear conditioning studies, may disguise dissociable influences of conditioned danger and safety signals, thereby disregarding distinct neural correlates (Fullana et al., 2015) which appear to be uniquely involved in pain-related emotional learning and memory processes (Benson et al., 2014; Gramsch et al., 2014; Icenhour et al., 2015a,b; Labrenz et al., 2015). Likewise, the ongoing debate regarding the putative impact of contingency awareness on different outcome measures of fear conditioning has widely neglected safety learning (e.g., Klucken et al., 2009; Schultz and Helmstetter, 2010; Lovibond et al., 2011; Tabbert et al., 2011). Our behavioral findings strongly support previous conclusions that safety-related learning processes deserve more attention in the context of pain (Vlaeyen, 2015) and in the broader field of fear conditioning (Fullana et al., 2015) and particularly encourage that contingency awareness does not only shape danger but also safety learning. Future research is clearly needed to extend these behavioral findings, for example by testing distinct effects of contingency accuracy on approach/avoidance behaviors. Additionally, broader methodological approaches including psychophysiological measures and functional brain imaging appear essential in light of previous reports supporting independent effects of contingency awareness on neural, psychophysiological and evaluative responses during classic fear conditioning in instructed aware and unaware subjects (Klucken et al., 2009; Tabbert et al., 2011). Finally, given evidence that contingency learning is altered in chronic pain (Jenewein et al., 2013; Meulders et al., 2014; Icenhour et al., 2015b), the results reported certainly call for further investigation to clarify the putative role of contingency awareness in impaired extinction in chronic pain.

## Author Contributions

AI, FL, SE designed the research studies. AI, FL performed the research. AI, FL, SB analyzed the data. AI, FL drafted the manuscript. All authors contributed to the interpretation of data, revised the manuscript and approved the final version of the manuscript.

## Funding

This re-analysis was based on two studies supported by a grant from the German Research Foundation (DFG, FOR1581 “Extinction Learning”).

## Conflict of Interest Statement

The authors declare that the research was conducted in the absence of any commercial or financial relationships that could be construed as a potential conflict of interest.
